# Towards Endoscopic No-Touch Saphenous Vein Graft Harvesting in Coronary Bypass Surgery

**DOI:** 10.21470/1678-9741-2022-0144

**Published:** 2022

**Authors:** Tomislav Kopjar, Michael R. Dashwood

**Affiliations:** 1 Department of Cardiac Surgery, School of Medicine, University of Zagreb, Zagreb, Croatia; 2 Surgical and Interventional Sciences, Royal Free Hospital Campus, University College London Medical School, United Kingdom

**Keywords:** Coronary Artery Bypass, Mamary Arteries, Saphenous Vein, Surgical Wound Infection, Infections.

## Abstract

The saphenous vein is the most used conduit for coronary artery bypass surgery. However, the patency rate of this graft is inferior to the internal thoracic artery patency rate, which is the gold standard. Using the conventional technique, the saphenous vein is harvested via a large open incision and excised in such a way that causes both vascular damage and wound healing complications. Consequently, vein graft patency and surgical site infection may be compromised. Graft patency is markedly improved when the saphenous vein is harvested atraumatically with minimal damage and with surrounding cushion of perivascular fat intact. However, despite the improved graft performance, wound healing complications and infection remain a problem. Although wound healing complication is reduced when using endoscopic vein harvesting, there may be a negative impact on graft performance. This is due to vascular damage associated with application of forces to the vein that are usually avoided in open vein harvesting, including traction, adventitial stripping, and venous compression. There is evidence to suggest that improved patency of endoscopically harvested saphenous veins is associated with the surgeon’s experience of the technique. Recently, endoscopic methods of harvesting have been described where the saphenous vein is removed intact and with minimal vascular damage caused. In addition, wound healing complications, infection, and scarring are reduced. While the effect of these techniques on vein graft patency have yet to be reported, the ability to obtain a superior graft with reduced wound complications will be of great benefit to patients undergoing coronary revascularization procedures.

**Table:** 

Abbreviations, Acronyms & Symbols			
BK	= Bradykinin	NOS	= Nitric oxide synthetase
CABG	= Coronary artery bypass grafting	NT	= No-touch
CT	= Closed tunnel	OT	= Open tunnel
EDR	= Endothelial-dependent relaxation	OVH	= Open harvesting
ESC/EACTS	= European Society of Cardiology/European Association for Cardio-Thoracic Surgery	PVAT REGROUP	= Perivascular adipose tissue = Randomized Endovein Graft Prospective
EVH	= Endoscopic vein harvesting	SNP	= Sodium nitroprusside
ITA	= Internal thoracic artery	SV	= Saphenous vein
L	= Lumen	SVG	= Saphenous vein graft
NICE	= National Institute for Health and Care Excellence	VEGF	= Vascular endothelial growth factor
NO	= Nitric oxide	VICO	= Vein Integrity and Clinical Outcomes

## INTRODUCTION

Although the saphenous vein (SV) is the most used conduit for cardiac revascularization in patients undergoing coronary artery bypass grafting (CABG), its performance is inferior to the performance of the internal thoracic artery (ITA)^[^1^-^4^]^ and, according to some, of the radial artery^[^5^-^7^]^. The SV was introduced as a graft over 50 years ago by Favaloro (1968), and, according to the methods, “Care must be taken to dissect only the vein, avoiding as much as possible the adventitia that surrounds it”. When preparing the SV in this manner, the cushion of surrounding fat is removed, and the adventitia is damaged^[^8^]^. In addition, the media, intima, and endothelium are damaged during vein harvesting due to a combination of vascular trauma and high-pressure intraluminal distention^[^8^,^9^]^. Favaloro’s method has been adopted as the favored, “conventional” technique where the SV is prepared by open harvesting (OVH) via a large incision made in the thigh or calf, a procedure causing scarring as well as wound complications in some patients^[^10^,^11^]^. Over 20 years ago, in an attempt to reduce these complications, the technique of endoscopic vein harvesting (EVH) was introduced^[^12^]^ where the SV is removed, generally via two small incisions of approximately 5 mm above the knee and a small space created for introduction of the endoscope. Carbon dioxide insufflation is often used to create a subcutaneous tunnel allowing for an easier separation from surrounding tissue, reducing bleeding and facilitating visualization. Once removed, the vein is flushed and distended, again at high pressure, to visualize side branches and leakage. Clearly, EVH requires forces to be applied to the vein that are usually avoided in OVH or no-touch (NT) vein harvesting, including traction, adventitial stripping, and venous compression, conditions that may cause considerable vessel damage^[^11^]^. EVH has been adopted by cardiac surgeons worldwide, particularly in the United States of America, where it was used in approximately 80% of all CABG procedures in 2005^[^13^]^. A number of studies/trials have been performed comparing the effect of EVH *versus* OVH with conflicting reports regarding the effect of EVH on graft patency. To date, only a few short- and mid-term follow-up trials comparing EVH and OVH patency have been performed with the general consensus being that patency of EVH grafts is, at best, comparable to OVH grafts^[^11^]^. Indeed, in the most recent Randomized Endovein Graft Prospective (REGROUP) trial, clinical outcomes of open or endoscopic vein-graft harvesting in CABG were assessed^[^14^]^. The REGROUP trial, a multicenter, randomized trial on a total of 1,150 patients, concludes “…we did not find a significant difference between open vein-graft harvesting and endoscopic vein-graft harvesting in the risk of major adverse cardiac events”^[^14^]^. As mentioned previously, vascular damage may be caused to EVH SVs used in CABG, a damage that may impact on graft performance.

## DISCUSSION

### Vascular Damage

**Fig. 1:**
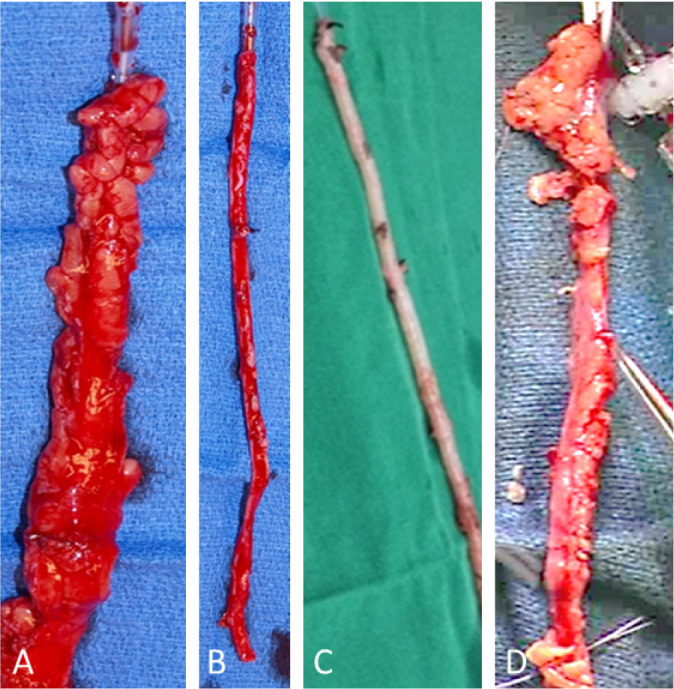
Comparison of saphenous veins harvested for coronary artery bypass grafting. Examples of saphenous vein explants at harvesting: A) No-touch, B) conventional, C) endoscopic, and D) no-touch endoscopic. (From Yoshino et al.^[^61^]^, 2020).

While SVs removed by both EVH and OVH have the outer pedicle removed and are subjected to varying degrees of damage, an atraumatic NT technique has been described (Figure 1), where the vein is removed completely with its cushion of surrounding fat intact^[^15^]^, and that provides an SV graft (SVG) with a patency superior to OVH SVs^[^16^]^ and comparable to the ITA^[^17^,^18^]^. Based on the excellent (> 80% after 16 years) long-term patency rates of the NT SVG shown in multiple randomized trials, the 2018 European Society of Cardiology/European Association for Cardio-Thoracic Surgery (ESC/EACTS) Guidelines on myocardial revascularization suggest its use whenever the OVH technique is used for SV harvesting in CABG. This was set as a Class IIa recommendation with the Level of evidence B^[^19^]^.

Since using NT harvesting, the SV is not handled directly by surgical instruments but via its cushion of fat, the vein does not go into spasm, high pressure distension is not required, and the luminal endothelium is mainly preserved^[^20^,^21^]^. The damage to NT SV is minimal when compared to that caused to OVH SVs, and the few studies reported on EVH SVs with NT SVs essentially maintaining a normal architecture^[^8^,^9^]^. These observations are suggested to explain the superior performance of NT SVG, since damage to various structures and the effect on various tissue- and cell-derived factors that are caused when using OVH do not occur or are minimized using NT harvesting. Such structures include the vasa vasorum^[^22^,^23^]^, the endothelium^[^9^,^21^,^24^]^, and vascular smooth muscle cells^[^24^,^25^]^. More recently, the role of perivascular adipose tissue (PVAT) on graft performance has attracted considerable attention, particularly via the so-called adipocyte-derived relaxing factor(s)^[^26^,^27^]^. While quite dramatic relaxant or anti-contractile effects of PVAT have been demonstrated in SVs harvested by NT *vs.* OVH SVs^[^28^]^, we believe that the recent study by Yamada et al. ^[^29^]^ is the first to describe a comparison between OVH and EVH SVs.

Since the introduction of EVH, this technique of preparing the SV for CABG has become widespread with over 80% of patients in the United States of America undergoing this form of harvesting^[^13^]^. While there is no doubt of the benefits of EVH regarding improved wound healing and reduced wound infection, there is some concern over the effect this procedure has on SV structure and the potential effect on graft patency. In fact, previous guidance in the United Kingdom advised that EVH should only be used with special arrangements^[^30^]^. However, a more recent National Institute for Health and Care Excellence (NICE) advice is that “Current evidence on the efficacy and safety of endoscopic saphenous vein harvest for coronary artery bypass grafting (CABG) is adequate to support the use of this procedure provided that normal arrangements are in place for clinical governance, consent and audit” (NICE 2014).

**Fig. 2:**
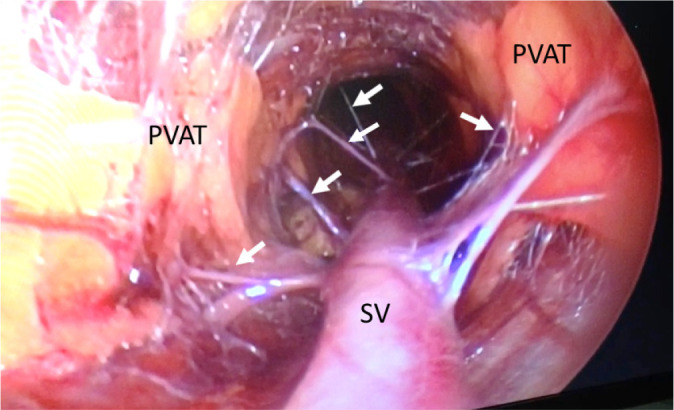
Conventional endoscopic saphenous vein (SV) graft harvesting for coronary artery bypass grafting. Screen shot from video footage taken during endoscopic SV harvesting showing exposure of the SV, separation of perivascular adipose tissue (PVAT), and vasa vasorum (arrows). (From Dashwood et al.^[^62^]^, 2020).

**Fig. 3:**
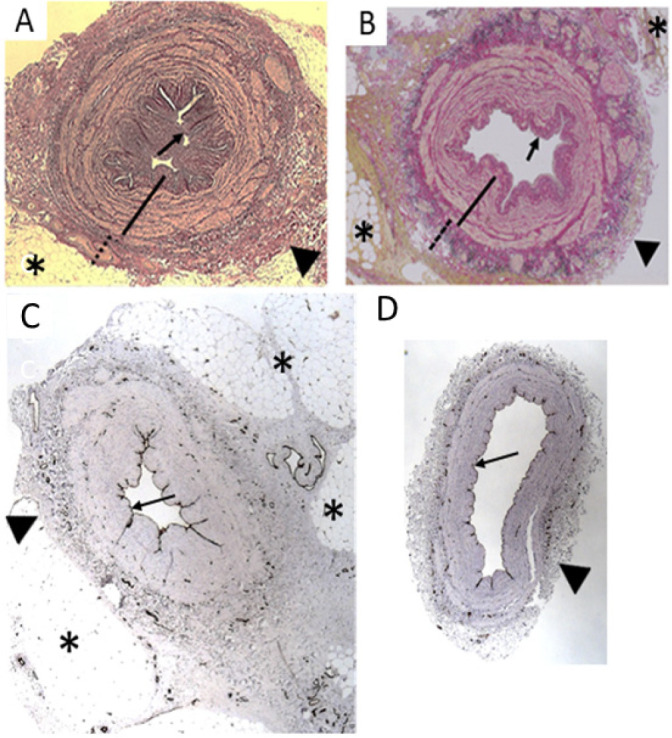
Histological appearance of saphenous vein grafts prepared using different techniques of harvesting. Top sections stained for muscle layers. A) Transverse section of a no-touch saphenous vein graft with perivascular adipose tissue. (From Dashwood et al.^[^9^]^, 2009). B) Transverse section of endoscopic no-touch saphenous vein graft with perivascular adipose tissue intact. (From Yoshino et al.^[^61^]^, 2020). C) No-touch saphenous vein with intact luminal endothelium, adventitia, and perivascular fat. (From Dashwood et al.^[^8^]^, 2013). D) Conventional saphenous vein with endothelium and adventitia damaged and perivascular adipose tissue removed. (From Dashwood et al.^[^8^]^, 2013). *=perivascular adipose tissue; arrowhead=adventitia; arrow=luminal endothelium.

A greater degree of damage to SVs harvested by EVH would be expected since this technique requires forces to be applied to the vein that are usually avoided in OVH or NT vein harvesting, including traction, adventitial stripping, and venous compression^[^11^]^. In the past, very few examples of damage to EVH SVs were available in the literature but more have appeared more recently. Clearly, like SVs harvested by OVH, the perivascular cushion of fat is removed (Figure 2) when using EVH. In general, most studies that have examined structural changes in SVs removed in this fashion have identified considerable damage to various regions (Figure 3), including the adventitia, intima, and endothelium^[^31^-^33^]^, although data from the Vein Integrity and Clinical Outcomes (VICO) Randomized Clinical Trial suggests that damage is minimal^[^34^]^. The VICO trial is the first study to directly evaluate the impact of minimally invasive and OVH techniques on the collective outcomes of endothelial integrity of the graft, clinical outcomes, health-related quality of life, and cost-effectiveness. This study compared OVH (*e.g.*, conventional) SVs with those harvested by closed tunnel (CT) and open tunnel (OT) EVH obtained from 300 CABG patients at 100 patients per group. Here, the OVH group demonstrated better endothelial integrity in random samples (85% *vs.* 88% *vs.* 93% for CT EVH, OT EVH, and OVH, respectively; *P*<0.001). However, there were no differences in endothelial stretching between groups. In total 2,700 SV samples were used and coded to ensure assessor blinding. Different groups (n=900) were studied comparing proximal SVs that were non distended, distal SVs flushed with 10 mmHg heparinized saline, and “random samples” from the remaining excised conduit. Thus, the three groups were suggested to represent “the entire vein at different stages after harvesting that could be achieved given the logistics of the operation”. While assessment was performed on a large number of SV sections, only four representative examples are illustrated showing varying degrees of endothelial disruption that was graded on a scale of 0 to 3 (normal *vs.* mild, moderate, or severe). While these results are presented in a rather confusing fashion, the SV sections shown appear to indicate that the degree of endothelial damage may be associated with a more generalized vascular damage. For example, the SV lumen of the section with an intact endothelium exhibits folds similar to those harvested by the NT technique (Figure 3) where no distension is used. The lumen of those sections with varying degrees of endothelial disruption is distended, indicating the use of pressure, either at harvesting or during histological processing (*i.e.*, similar to conventional SV where high pressure distension is used). In addition, pronounced patches of CD34 immunostaining are present at the adventitial/medial border, presumably of the vasa vasorum. The examples shown also indicate that most, if not all, of the perivascular fat has been removed. This study was an extension/follow-up from the same group published two years previously comparing EVH and OVH SV harvesting where endothelial detachment was significantly greater in the OVH than either the CT or OT endoscopic groups^[^33^]^. However, this study was performed on a small number of patients, and the histological findings should be interpreted with caution. The authors did not examine the vasomotor function of the SVG.

Apart from the histological data, as previously mentioned, the study of Krishnamoorthy et al. provides an interesting cost-effective analysis^[^34^]^. The costs of both the endoscopic approaches were higher than for traditional OVH, with CT EVH increasing costs by £1180 and OT EVH increasing costs by £981 per patient over OVH. However, there was a reduction in postoperative costs for EVH, CT EVH led to a mean reduction in costs of £814 per patient *vs.* OVH, whereas OT EVH led to a mean reduction of £598. Overall, when harvesting cost and downstream costs were combined, both EVH methods led to net cost increases over OVH, although neither was statistically significant. In conclusion, the authors state that harvesting techniques affect the integrity of different vein layers, albeit only slightly, and those histological findings do not directly contribute to major adverse cardiac event. Furthermore, high-level experience with endoscopic harvesting performed by a dedicated specialist practitioner gives optimal results comparable to those of OVH.

Any vascular damage will affect a variety of tissue- and cell-derived factors, impacting on various aspects of SVG performance including platelet aggregation, vascular smooth muscle cell proliferation, neointimal hyperplasia, and vasoreactivity^[^8^,^35^]^.

### Vascular Function

In the last 30 years, a number of *in vitro* studies examining the vasoreactivity of conduits used in CABG have been published^[^36^-^38^]^ with many recently focusing on the potential role of perivascular fat^[^28^,^29^,^39^]^. While these studies have shown varied effects of perivascular fat on OVH *vs.* NT SV segments *in vitro*, as far as we are aware, only one recent study has used segments of SVs removed by EVH^[^40^]^. Here, nitric oxide (NO)-mediated endothelial-dependent relaxation (EDR) in vein segments harvested for lower extremity bypass using open surgical techniques was compared with that with EVH techniques. Endothelial dependent relaxation was determined using bradykinin (BK), and endothelial-independent relaxation was confirmed using sodium nitroprusside (SNP). Mean percent relaxation for BK concentration showed a statistically significant improved EDR in EVH samples compared with OVH SVs and mean nitrite/nitrate tissue bath concentration measurements post-BK were significantly higher in EVH *vs.* OVH SVs. In addition, Factor VIII immunohistochemistry staining showed that endothelial integrity was preserved and was similar in both the EVH and OVH groups. Taken together it was concluded that endothelial function is preserved when using EVH, and that the advantages of minimally invasive vein procurement for lower extremity bypass can be obtained without concern for damaging venous endothelium. While SVs in this study were used as lower extremity grafts, the histological data is in general agreement with similar studies where SVs were used in CABG, suggesting that endothelial integrity is similar whether SVs are harvested by OVH or EVH^[^40^]^.

Conflicting data from PVAT/SV organ bath studies have been reported with some suggesting PVAT to possess anti-contractile actions and with others suggesting that PVAT-derived factor(s) are contractile^[^29^]^. This study was on small patient numbers, using myography, showing that contractions to phenylephrine were greater in NT SVs than in OVH SVs, and that this effect was “eliminated” when SVs were harvested using electrocautery. Here, NT SVs were used in organ bath studies where percent EDR to BK was “similar” between NT and OVH SV. When using the NO synthetase (NOS) inhibitor L-NAME, endothelium/NO-dependent relaxation in NT *vs.* OVH was said to be “equivalent”. Furthermore, there was more contraction at lower concentrations of SNP in the NT group, and relaxation at higher concentrations of SNP, when compared to OVH SV. This group also used endothelial NOS immunohistochemistry to assess SV stimulation by vascular endothelial growth factor (VEGF) at different time periods after harvesting. Densitometric analysis was used to determine the response to VEGF where the NT group was significantly better than the conventional OVH group at five and 60 minutes. Interestingly, as reported by others, by exchanging organ bath medium, the authors provide evidence for a transferable anti-contractile effect of PVAT^[^29^]^. Based on their data, this group concluded that “the NT technique is suggested to be advantageous for preserving the functions of vasoconstriction and relaxation”. Also, it was suspected that PVAT maintains vascular tone by releasing vasoconstrictive factors. However, in both studies by Wheeler et al.^[^40^]^ and Yamada et al.^[^29^]^, patient/SV segment numbers are low, and illustrations of SV histology indicate vascular damage to veins has occurred and are of poor quality.

There is a consensus that PVAT releases anti-contractile factors, based on early studies performed 30 years ago^[^41^]^ and discussed in a number of review articles^[^26^,^27^,^42^-^44^]^. While many studies into the effects of PVAT have been performed in rats or other experimental species^[^41^,^45^,^46^]^, there are those that have been performed specifically on vessels used as bypass conduits in CABG. In general, these studies focus on the two main vessels used for myocardial revascularization, ITA^[^39^,^47^]^ and SV^[^26^-^28^,^48^]^. Apart from their anti-contractile properties, certain PVAT-derived factors may possess additional actions beneficial for graft performance. For example, NOS has been identified in PVAT of NT SVG sections with tissue extracts exhibiting the ability to generate NO^[^9^]^. Preserving PVAT was predominantly involved in the superior nitrogen oxides production in NT when compared to conventional SVG^[^49^]^. The preservation of this source of NO potentially contributes to reducing spasm at harvesting and vasoconstriction post implantation as well as preventing platelet aggregation, thrombus formation, and neointimal hyperplasia, processes underlying both early- and late-stages of graft occlusion^[^9^,^26^,^27^]^. Since NO plays crucial roles in suppressing atherosclerosis, this mechanism may greatly contribute to the excellent patency in NT SVG. In addition to its vasoactive properties, PVAT also has a mechanical role in improving SVG performance where this external cushion not only protects the graft against the effects of increased coronary artery hemodynamics, but also provides support and prevents kinking in grafts of excessive length^[^26^,^27^,^50^,^51^]^. This natural property of PVAT appears not to have been considered, or has been overlooked, since there have been various strategies aimed at replacing the cushion of surrounding fat that is removed when using conventional OVH. For example, this prominent outermost vessel layer not only prevents the SV from going into spasm at harvesting but also protects the endothelium against intraluminal pressures of 300 mmHg^[^8^,^9^]^. Various artificial methods of providing artificial support to conventional SVs have been studied, ranging from the use of a monofilament knitted tube^[^52^]^ and fibrin glue^[^53^]^ to “extents” made of Dacron^[^54^]^ and of braided cobalt-chromium-nickel-molybdenum-iron alloy fibers^[^51^,^55^]^. The rationale for using external stents on damaged conventional SVG ranges from providing mechanical support to protection against the effect of arterial hemodynamics and the stimulation of angiogenesis^[^51^]^. One might question the reason for introducing such strategies that may be technically challenging, costly, and potentially harmful to patients undergoing CABG. For example, the external stent that showed such promise in an experimental pig model proved disastrous in the Extent trial where all extent SVG were thrombosed, but non-stented SV and internal mammary artery grafts remained patent^[^56^]^.

### Leg Wound Healing

The three most used techniques for harvesting the SV today are completely open, bridged, and endoscopic techniques. In OVH, the SV is exposed using extensive skin incisions thereby providing superior access and visualization of the SV. However, with OVH, there is an increased risk of wound complications and postoperative pain. The bridged technique involves performing two or three step incisions over the course of the vein, dissecting as in OVH but with branches divided *in situ* and ligated once the SV is explanted. Endoscopic vein harvesting is a minimally invasive technique where the SV is explanted through a small incision on the skin resulting in reduced postoperative morbidity and improved patient satisfaction.

Mainly, there are two commercially available systems for EVH. A CT system, also known as a sealed system, occludes the access site with a balloon and insufflates the dissection tunnel with CO_2_ at up to 12-mmHg pressure. The OT system, also known as a non-sealed system, does not occlude the access site or pressurize the dissection tunnel. Both systems allow for a clear vein visualization, mobilization, and branch ligation. The vein branches can be either clipped or cauterized. For either system, in EVH, a small incision is made just above or below the knee depending on the length of vein required for surgery. The endoscope is usually equipped with a sharp, clear dissecting cone on the tip, or a blunt spoon-like retractor. It is inserted through the skin incision. After a few centimeters of anterior dissection, the balloon is inflated to seal the incision port in the CT system. The vein is dissected from the surrounding tissues anteriorly and posteriorly until reaching the femoral junction in the groin. The vein side branches are usually ligated or clipped once removed from the leg. Endoscopic vein harvesting is associated with reduced scarring and postoperative pain, reduced inflammation and infection, and greater patient mobility^[^34^]^. If performed by experienced surgeons, it should be considered to reduce the incidence of wound complications. This is a Class IIa, Level A recommendation from the 2018 ESC/EACTS Guidelines on myocardial revascularization^[^19^]^.

The excision of surrounding tissues and the creation of skin flaps by the NT technique of SV harvesting are commonly debated to lead to more extensive tissue damage. Studies have reported a higher rate of SV harvesting site infection in patients receiving the NT technique^[^16^,^57^,^58^]^. These rates vary between studies from about 10 to 25%. The NT technique requires more meticulous intraoperative incision closure and postoperative wound management. However, these wound complications are mostly mild and less likely to affect long-term life function or quality^[^16^,^25^]^.

**Fig. 4:**
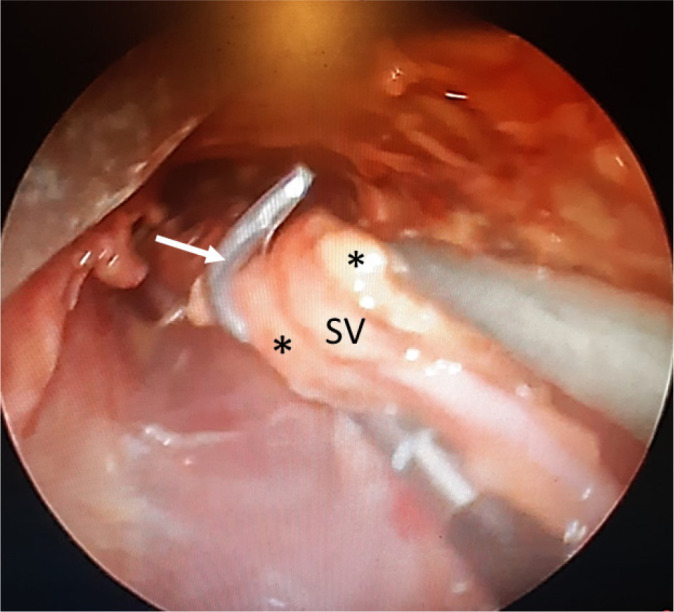
No-touch endoscopic saphenous vein (SV) graft harvesting. Screen shot from a video footage taken at harvesting where perivascular adipose tissue (*) remains intact surrounding the SV. (From Hayashi et al.^[^60^]^, 2020).

**Fig. 5:**
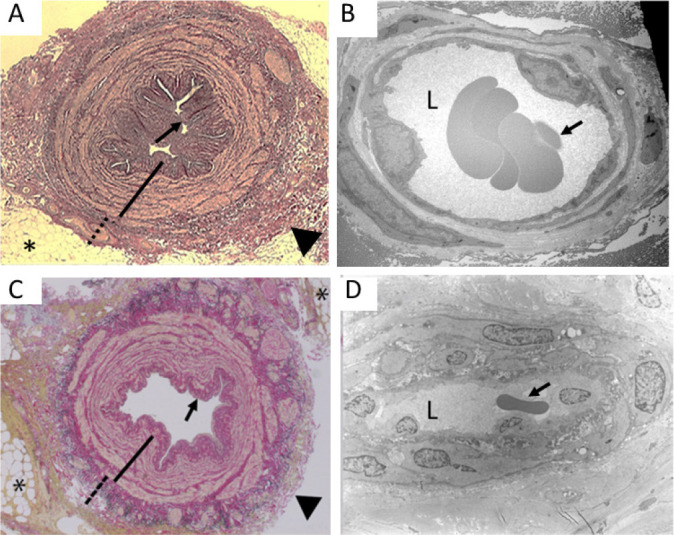
Histology findings of no-touch and endoscopic saphenous vein grafts for coronary artery bypass grafting. Left panels show representative transverse sections of no-touch (A) and endoscopic no-touch (C) saphenous vein grafts with preserved perivascular adipose tissue (*), a folded intima (small arrow), a thick intima (continuous line), and an intact/undamaged adventitia (broken line). Right panels show transmission electron microscopy images of open adventitial vasa vasorum lumen (L) containing erythrocytes (small arrow) in no-touch (B) and endoscopic no-touch (D) saphenous vein grafts. (A from Dashwood et al.^[^9^]^, 2009; B from Ahmed et al.^[^24^]^, 2004; C and D from Yoshino et al.^[^61^]^, 2020).

A recent study by Hayashi, Kashima, and Yoshikawa (2020) describes a technique, similar to NT SVG harvesting, employing an electrothermal bipolar vessel sealing device via small incisions^[^59^]^. The SVG was harvested with a pedicle of surrounding tissue approximately 5 mm in size intact and preserving a normal intima, media, adventitia, and vasa vasorum as confirmed by histological analysis. This technique is suggested to combine the potential advantages of minimally invasive endoscopic harvesting using bipolar electrothermy and the improved patency of NT SVG^[^59^]^. A more recent study by this group provides video footage demonstrating an endoscopic NT SVG technique (Figure 4) employing a reusable SV retractor system without CO_2_ insufflation and an electrothermal bipolar vessel sealing device. An initial ultrasonographic course mapping was used to evaluate unusable varicose or very small veins. A 3-cm incision was made in the upper knee, parallel to the vein, and a subcutaneous tunnel was created under videoendoscopic control. Endoscopic dissection of the NT SV and side branches was then performed using an electrothermal bipolar vessel sealing device^[^60^]^. Another study from a Japanese group also describes a form of endoscopic NT SV harvesting, where the SV is harvested complete with perivascular tissue intact. It is performed via a 3-cm skin incision made at the medial side of the thigh, just above the knee, using Vasoview Hemopro 2 Endoscopic Vessel Harvesting System (Getinge AB, Göteborg, Sweden)^[^61^]^. An important aspect of this study is that histological examinations of the unused portion of the SVG confirmed the preservation of perivascular tissue. Here, the histology revealed the appearance of intimal folding, the presence of perivascular connective tissue, and PVAT with electron microscopic examination (Figure 5) showing a patent vasa vasorum^[^61^]^. In the small number of patients in this study, none experienced surgical site infection and antibiotic treatment was not required, and any minor complications were short-lived.

Given the marked improvement in NT SVG patency and the reduced leg wound complications when using EVH, these recent studies may pave the way towards a greater use of NT SVG, securing its position as the second conduit of choice for CABG^[^62^]^.

## CONCLUSION

The SV is the most used conduit for revascularization in patients undergoing CABG. The patency of conventional SVG is affected by vascular damage caused at harvesting but is improved dramatically when the vein is prepared with minimal trauma using the NT SV harvesting technique. However, in both cases the SV is removed via large open incision, a situation leading to wound infection, wound healing problems, and scarring. These surgical site problems are overcome using EVH, where the SV is harvested through small incisions using specialized instruments under video control. The limited visual field and other conditions, such as traction and handling by instruments associated with EVH, may cause damage to the SV, a damage that affects graft performance. There is an overall shortage of properly designed prospective randomized studies comparing long-term graft performance of EVH vein grafts. Recently, a number of modified EVH procedures have been introduced that protect SV structure and reduce wound healing complications, infection, and scarring. To date, no follow-up patient studies have been reported using these EVH NT SVG techniques. It is important for such trials to be conducted to determine their effectiveness in producing superior SVG for CABG with minimal surgical site problems.

**Table:** 

Authors’ Roles & Responsibilities	
TK	Substantial contributions to the conception or design of the work; or the acquisition, analysis, or interpretation of data for the work; drafting the work or revising it critically for important intellectual content; agreement to be accountable for all aspects of the work in ensuring that questions related to the accuracy or integrity of any part of the work are appropriately investigated and resolved; final approval of the version to be published
MRD	Substantial contributions to the conception or design of the work; or the acquisition, analysis, or interpretation of data for the work; drafting the work or revising it critically for important intellectual content; agreement to be accountable for all aspects of the work in ensuring that questions related to the accuracy or integrity of any part of the work are appropriately investigated and resolved; final approval of the version to be published

## References

[1] Schwann TA, Habib RH, Wallace A, Shahian DM, O'Brien S, Jacobs JP (2018). Operative outcomes of multiple-arterial versus single-arterial
coronary bypass grafting. Ann Thorac Surg.

[2] Fitzgibbon GM, Kafka HP, Leach AJ, Keon WJ, Hooper GD, Burton JR. (1996). Coronary bypass graft fate and patient outcome: angiographic
follow-up of 5,065 grafts related to survival and reoperation in 1,388
patients during 25 years. J Am Coll Cardiol.

[3] Loop FD, Lytle BW, Cosgrove DM, Stewart RW, Goormastic M, Williams GW (1986). Influence of the internal-mammary-artery graft on 10-year
survival and other cardiac events. N Engl J Med.

[4] Taggart DP, Altman DG, Gray AM, Lees B, Gerry S, Benedetto U (2016). Randomized trial of bilateral versus single
internal-thoracic-artery grafts. N Engl J Med.

[5] Tatoulis J, Buxton BF, Fuller JA, Meswani M, Theodore S, Powar N (2009). Long-term patency of 1108 radial arterial-coronary angiograms
over 10 years. Ann Thorac Surg.

[6] Gaudino M, Taggart D, Suma H, Puskas JD, Crea F, Massetti M. (2015). The choice of conduits in coronary artery bypass
surgery. J Am Coll Cardiol.

[7] Gaudino M, Mack MJ, Taggart DP. (2018). Additional arterial conduits in coronary artery bypass surgery:
finally coming of age. J Thorac Cardiovasc Surg.

[8] Dashwood MR, Tsui JC. (2013). 'No-touch' saphenous vein harvesting improves graft performance
in patients undergoing coronary artery bypass surgery: a journey from
bedside to bench. Vascul Pharmacol.

[9] Dashwood MR, Savage K, Tsui JC, Dooley A, Shaw SG, Fernández Alfonso MS (2009). Retaining perivascular tissue of human saphenous vein grafts
protects against surgical and distension-induced damage and preserves
endothelial nitric oxide synthase and nitric oxide synthase
activity. J Thorac Cardiovasc Surg.

[10] Kodia K, Patel S, Weber MP, Luc JGY, Choi JH, Maynes EJ (2018). Graft patency after open versus endoscopic saphenous vein harvest
in coronary artery bypass grafting surgery: a systematic review and
meta-analysis. Ann Cardiothorac Surg.

[11] Kopjar T, Dashwood MR. (2016). Endoscopic versus "no-touch" saphenous vein harvesting for
coronary artery bypass grafting: a trade-off between wound healing and graft
patency. Angiology.

[12] Lumsden AB, Eaves FF (1996). 3rd, Ofenloch JC, Jordan WD. Subcutaneous, video-assisted
saphenous vein harvest: report of the first 30 cases. Cardiovasc Surg.

[13] Dacey LJ, Braxton JH, Kramer RS, Schmoker JD, Charlesworth DC, Helm RE (2011). Long-term outcomes of endoscopic vein harvesting after coronary
artery bypass grafting. Circulation.

[14] Zenati MA, Bhatt DL, Bakaeen FG, Stock EM, Biswas K, Gaziano JM (2019). Randomized trial of endoscopic or open vein-graft harvesting for
coronary-artery bypass. N Engl J Med.

[15] Souza D. (1996). A new no-touch preparation technique. Technical
notes. Scand J Thorac Cardiovasc Surg.

[16] Tian M, Wang X, Sun H, Feng W, Song Y, Lu F (2021). No-touch versus conventional vein harvesting techniques at 12
months after coronary artery bypass grafting surgery: multicenter
randomized, controlled trial. Circulation.

[17] Souza DS, Johansson B, Bojö L, Karlsson R, Geijer H, Filbey D (2006). Harvesting the saphenous vein with surrounding tissue for CABG
provides long-term graft patency comparable to the left internal thoracic
artery: results of a randomized longitudinal trial. J Thorac Cardiovasc Surg.

[18] Samano N, Geijer H, Liden M, Fremes S, Bodin L, Souza D. (2015). The no-touch saphenous vein for coronary artery bypass grafting
maintains a patency, after 16 years, comparable to the left internal
thoracic artery: a randomized trial. J Thorac Cardiovasc Surg.

[19] Sousa-Uva M, Neumann FJ, Ahlsson A, Alfonso F, Banning AP, Benedetto U (2019). 2018 ESC/EACTS guidelines on myocardial
revascularization. Eur J Cardiothorac Surg.

[20] Souza DS, Christofferson RH, Bomfim V, Filbey D. (1999). "No-touch" technique using saphenous vein harvested with its
surrounding tissue for coronary artery bypass grafting maintains an intact
endothelium. Scand Cardiovasc J.

[21] Tsui JC, Souza DS, Filbey D, Bomfim V, Dashwood MR. (2001). Preserved endothelial integrity and nitric oxide synthase in
saphenous vein grafts harvested by a 'no-touch' technique. Br J Surg.

[22] Dashwood MR, Anand R, Loesch A, Souza DS. (2004). Hypothesis: a potential role for the vasa vasorum in the
maintenance of vein graft patency. Angiology.

[23] Dreifaldt M, Souza DS, Loesch A, Muddle JR, Karlsson MG, Filbey D (2011). The "no-touch" harvesting technique for vein grafts in coronary
artery bypass surgery preserves an intact vasa vasorum. J Thorac Cardiovasc Surg.

[24] Ahmed SR, Johansson BL, Karlsson MG, Souza DS, Dashwood MR, Loesch A. (2004). Human saphenous vein and coronary bypass surgery: ultrastructural
aspects of conventional and "no-touch" vein graft
preparations. Histol Histopathol.

[25] Verma S, Lovren F, Pan Y, Yanagawa B, Deb S, Karkhanis R (2014). Pedicled no-touch saphenous vein graft harvest limits vascular
smooth muscle cell activation: the PATENT saphenous vein graft
study. Eur J Cardiothorac Surg.

[26] Fernandez-Alfonso MS, Souza DS, Dreifaldt M, Dashwood MR. (2016). Commentary: perivascular fat and improved vein graft patency in
patients undergoing coronary artery bypass surgery. Curr Vasc Pharmacol.

[27] Fernández-Alfonso MS, Gil-Ortega M, Aranguez I, Souza D, Dreifaldt M, Somoza B (2017). Role of PVAT in coronary atherosclerosis and vein graft patency:
friend or foe?. Br J Pharmacol.

[28] Ozen G, Topal G, Gomez I, Ghorreshi A, Boukais K, Benyahia C (2013). Control of human vascular tone by prostanoids derived from
perivascular adipose tissue. Prostaglandins Other Lipid Mediat.

[29] Yamada T, Adachi T, Ido Y, Masaki N, Toya T, Uchimuro T (2018). Preserved vasoconstriction and relaxation of saphenous vein
grafts obtained by a no-touch technique for coronary artery bypass
grafting. Circ J.

[30] Barnard JB, Keenan DJ, National Institute for Health and Clinical (2011). Endoscopic saphenous vein harvesting for coronary artery bypass
grafts: NICE guidance. Heart.

[31] Alrawi SJ, Balaya F, Raju R, Cunningham JN (2001). Jr, Acinapura AJ. A comparative study of endothelial cell injury
during open and endoscopic saphenectomy: an electron microscopic
evaluation. Heart Surg Forum.

[32] Kiani S, Poston R. (2011). Is endoscopic harvesting bad for saphenous vein graft patency in
coronary surgery?. Curr Opin Cardiol.

[33] Hashmi SF, Krishnamoorthy B, Critchley WR, Walker P, Bishop PW, Venkateswaran RV (2015). Histological and immunohistochemical evaluation of human
saphenous vein harvested by endoscopic and open conventional
methods. Interact Cardiovasc Thorac Surg.

[34] Krishnamoorthy B, Critchley WR, Thompson AJ, Payne K, Morris J, Venkateswaran RV (2017). Study comparing vein integrity and clinical outcomes in open vein
harvesting and 2 types of endoscopic vein harvesting for coronary artery
bypass grafting: the VICO randomized clinical trial (vein integrity and
clinical outcomes). Circulation.

[35] Tsui JC, Dashwood MR. (2002). Recent strategies to reduce vein graft occlusion: a need to limit
the effect of vascular damage. Eur J Vasc Endovasc Surg.

[36] Tadjkarimi S, O'Neil GS, Luu TN, Allen SP, Schyns CJ, Chester AH (1992). Comparison of cyclic GMP in human internal mammary artery and
saphenous vein: implications for coronary artery bypass graft
patency. Cardiovasc Res.

[37] Thorin-Trescases N, Dimitri WR, Dominiczak AF, Hamilton CA, Reid JL. (1993). Vasorelaxant properties of isolated human internal mammary
arteries and saphenous veins: comparative effects of milrinone and sodium
nitroprusside. J Cardiovasc Pharmacol.

[38] Allen SP, Chester AH, Dashwood MR, Tadjkarimi S, Piper PJ, Yacoub MH. (1994). Preferential vasoconstriction to cysteinyl leukotrienes in the
human saphenous vein compared with the internal mammary artery. Implications
for graft performance. Circulation.

[39] Malinowski M, Deja MA, Gołba KS, Roleder T, Biernat J, Woś S. (2008). Perivascular tissue of internal thoracic artery releases potent
nitric oxide and prostacyclin-independent anticontractile
factor. Eur J Cardiothorac Surg.

[40] Wheeler AR, Kendrick DE, Allemang MT, Gosling AF, Kim AH, Hausladen A (2017). Endothelial function is preserved in veins harvested by either
endoscopic or surgical techniques. Ann Vasc Surg.

[41] Soltis EE, Cassis LA. (1991). Influence of perivascular adipose tissue on rat aortic smooth
muscle responsiveness. Clin Exp Hypertens A.

[42] Gollasch M, Dubrovska G. (2004). Paracrine role for periadventitial adipose tissue in the
regulation of arterial tone. Trends Pharmacol Sci.

[43] Gollasch M. (2012). Vasodilator signals from perivascular adipose
tissue. Br J Pharmacol.

[44] Zaborska KE, Wareing M, Austin C. (2017). Comparisons between perivascular adipose tissue and the
endothelium in their modulation of vascular tone. Br J Pharmacol.

[45] Lee HJ, Cantú SM, Álvarez Primo M, Peredo HA, Donoso AS, Puyó AM (2021). Losartan prevents mesenteric vascular bed alterations in high-fat
diet fed rats. Clin Investig Arterioscler.

[46] Chang HH, Yang SS, Chang SJ. (2020). Perivascular adipose tissue modulation of neurogenic
vasorelaxation of rat mesenteric arteries. J Cardiovasc Pharmacol.

[47] Gao YJ, Zeng ZH, Teoh K, Sharma AM, Abouzahr L, Cybulsky I (2005). Perivascular adipose tissue modulates vascular function in the
human internal thoracic artery. J Thorac Cardiovasc Surg.

[48] Ford CA, Mong K, Tabrizchi R. (2006). Influence of tangential stress on mechanical responses to
vasoactive agents in human saphenous vein with and without perivascular
adipose tissue. Can J Cardiol.

[49] Saito T, Kurazumi H, Suzuki R, Matsunaga K, Tsubone S, Lv B (2022). Perivascular adipose tissue is a major source of nitric oxide in
saphenous vein grafts harvested via the no-touch technique. J Am Heart Assoc.

[50] Rueda Fd, Souza D, Lima Rde C, Menezes A, Johansson B, Dashwood M (2008). Novel no-touch technique of harvesting the saphenous vein for
coronary artery bypass grafting. Arq Bras Cardiol.

[51] Samano N, Souza D, Dashwood MR. (2021). Saphenous veins in coronary artery bypass grafting need external
support. Asian Cardiovasc Thorac Ann.

[52] Parsonnet V, Lari AA, Shah IH. (1963). New stent for support of veins in arterial grafts. Arch Surg.

[53] Stooker W, Niessen HW, Wildevuur WR, van Hinsbergh VW, Fritz J, Jansen EK (2002). Perivenous application of fibrin glue reduces early injury to the
human saphenous vein graft wall in an ex vivo model. Eur J Cardiothorac Surg.

[54] Violaris AG, Newby AC, Angelini GD. (1993). Effects of external stenting on wall thickening in arteriovenous
bypass grafts. Ann Thorac Surg.

[55] Ben-Gal Y, Taggart DP, Williams MR, Orion E, Uretzky G, Shofti R (2013). Expandable external support device to improve saphenous vein
graft patency after CABG. J Cardiothorac Surg.

[56] Murphy GJ, Newby AC, Jeremy JY, Baumbach A, Angelini GD (2007). A randomized trial of an external dacron sheath for the
prevention of vein graft disease: the extent study. J Thorac Cardiovasc Surg.

[57] Deb S, Singh SK, de Souza D, Chu MWA, Whitlock R, Meyer SR (2019). SUPERIOR SVG: no touch saphenous harvesting to improve patency
following coronary bypass grafting (a multi-centre randomized control trial,
NCT01047449). J Cardiothorac Surg.

[58] Souza DS, Dashwood MR, Tsui JC, Filbey D, Bodin L, Johansson B (2002). Improved patency in vein grafts harvested with surrounding
tissue: results of a randomized study using three harvesting
techniques. Ann Thorac Surg.

[59] Hayashi I, Kashima I, Yoshikawa E. (2020). Use of the no-touch saphenous vein harvesting technique via small
incisions. Innovations (Phila).

[60] Hayashi I, Kashima I, Yoshikawa E. (2020). The endoscopic no-touch saphenous vein harvesting
technique. Multimed Man Cardiothorac Surg.

[61] Yoshino K, Abe K, Suzuki K, Tamaki R, Mituishi A, Yamasaki M (2020). A novel technique of endoscopic vein harvesting with preserved
perivascular tissue. Innovations (Phila).

[62] Dashwood MR, Melo Silva HS, Lima ML. (2020). Endoscopic harvesting will secure a silver medal for the no-touch
saphenous vein and bronze for the radial artery. J Thorac Cardiovasc Surg.

